# OVATE family protein 6 interacts with CONSTANS-LIKE 3 to regulate bolting by transcriptional control of *FLOWERING LOCUS T*/*TERMINAL FLOWER 1* family genes in lettuce

**DOI:** 10.1093/hr/uhag225

**Published:** 2026-06-05

**Authors:** Guanghui An, Ruoqing Liu, Xinhui Zhao, Yueji Wang, Chenxi Li, Lei Zhu, Luming Yang, Peinan Sun, Yong Wang

**Affiliations:** College of Horticulture, Henan Agricultural University, Zhengzhou 450046, China; College of Horticulture, Henan Agricultural University, Zhengzhou 450046, China; College of Horticulture, Henan Agricultural University, Zhengzhou 450046, China; College of Horticulture, Henan Agricultural University, Zhengzhou 450046, China; College of Horticulture, Henan Agricultural University, Zhengzhou 450046, China; College of Horticulture, Henan Agricultural University, Zhengzhou 450046, China; College of Horticulture, Henan Agricultural University, Zhengzhou 450046, China; College of Life Science, Dezhou University, Dezhou 253023, China; College of Horticulture, Henan Agricultural University, Zhengzhou 450046, China

## Abstract

As one of the most globally cultivated vegetables, lettuce’s commercial yield is severely compromised by premature bolting, an undesirable agronomic trait that diminishes crop quality and market value. In this study, we genetically cloned the *OVATE family protein 6* (*LsOFP6*) controlling bolting time in lettuce. *LsOFP6* had a nonsense mutation in the late bolting parent. CRISPR/Cas9 knockout and complementary tests confirmed that *LsOFP6* positively regulates bolting in lettuce. LsOFP6 physically interacts with the CONSTANS-LIKE 3 (LsCOL3). Genetic analyses suggest that *LsOFP6* regulates bolting through an *LsCOL3*-dependent pathway in the *Lsblh2 Lskn1* genotype background, where we have previously established that LsBLH2 and LsKN1, the positive and negative transcriptional regulators of bolting, respectively, both interact with LsOFP6 and exert their functions via the gibberellin pathway. LsCOL3 represses bolting development by directly binding to the promoters of *TWIN SISTER OF FLOWERING LOCUS T* (*LsTSF1*) and *TERMINAL FLOWER 1* (*LsTFL1*), downregulating the former while upregulating the latter. These regulations are antagonized by LsOFP6, which promotes bolting by compromising the effects of LsCOL3 on *LsTSF1* and *LsTFL1*. Therefore, loss of function of *Lsofp6* decreases *LsTSF1* and increases *LsTFL1* expression, resulting in late bolting. In summary, the LsOFP6–LsCOL3 module regulates lettuce bolting development by controlling the transcript levels of *LsTSF1* and *LsTFL1* genes. This study deepens the molecular understanding of bolting development and provides valuable genetic resources and novel strategies for developing lettuce with improved resistance to premature bolting, highlighting the power of natural variation to address agricultural challenges.

## Introduction

Lettuce (*Lactuca sativa*) is a globally significant vegetable crop and is primarily cultivated for its edible rosette leaves. Lettuce has also been genetically modified to serve as a bioreactor for producing protein-based therapeutics, including high-value pharmaceutical proteins and edible vaccines [1]. Premature bolting, an undesirable agronomic trait, severely limits vegetative biomass accumulation, impairs crop quality, and leads to yield reduction, thereby causing considerable economic losses in lettuce production [2, 3]. As a major challenge facing both growers and the vegetable industry, elucidating the genetic components and regulatory networks underlying bolting development is essential for breeding lettuce varieties with enhanced resistance to this phenomenon.

OVATE family proteins (OFPs) function as plant-specific transcriptional regulators distinguished by a conserved C-terminal OVATE domain. They play pivotal roles in diverse aspects of plant growth and development process, such as plant architecture, organ size, and fruit shape [4–9]. For instance, AtOFP1 overexpression suppresses cell elongation, resulting in rounded seeds and curled leaves in *Arabidopsis* [10]; OsOFP8/9/14 integrate multiple hormonal pathways to regulate key traits in rice by interacting with partners like Grain Shape Gene on Chromosome 9 (GS9) or DWARF AND LOW-TILLERING (DLT) [6, 11]. OFPs interact with TONNEAU1 Recruiting Motif proteins (TRMs) to impact organ shape along both the mediolateral and proximo-distal axes of tomato growth [9]. A central mechanism underlying OFP function is their interaction with three-amino-acid-loop-extension homeodomain proteins (TALEs) [including BELL-like homeodomain (BLHs) and KNOTTED1-like homeobox (KNOXs) families], forming a critical regulatory network for plant development [12]. Our earlier work established that LsOFP6 affects bolting via the LsBLH2- and LsKN1-mediated transcriptional control of gibberellin (GA) biosynthesis and signaling genes [2, 3]. However, it remains unclear whether LsOFP6 regulates bolting solely through the LsBLH2- and LsKN1-mediated GA pathway. Additionally, if LsOFP6 influences bolting via alternative mechanisms, the underlying genetic and molecular basis of such pathways has yet to be elucidated.

The CCT (CONSTANS, CO-like, and TOC1) gene family represents a crucial class of transcriptional regulators in plants, playing multifaceted roles in the control of flowering time, growth and development, and responses to biotic and abiotic stresses [13–16]. Among these, the CO-like genes (COLs) form a highly conserved subfamily across both monocots and dicots, serving as central regulators of bolting and flowering [17, 18]. COLs are distinguished by the presence of one or two B-box zinc finger domains at their N-terminus, which facilitate interactions between proteins, and a C-terminal CCT domain implicated in both protein interaction and nuclear localization, with potential DNA-binding activity [18, 19]. COLs integrate environmental cues, particularly photoperiod, into developmental programs, thereby optimizing bolting and flowering time [20]. Their conserved structure and functional significance across plant species underscore their importance in adaptation and crop improvement. However, COLs have not yet been characterized in lettuce, and the precise biochemical mechanisms through which COLs control the expression of their target genes are still not well understood.

The *FLOWERING LOCUS T*/*TERMINAL FLOWER 1* (*FT*/*TFL1*) family genes, encoding phosphatidylethanolamine-binding proteins (PEBPs), serve as integrators for signals from various flowering pathways and play a crucial role in the transition from vegetative growth to reproductive development in many crop species, exhibiting substantial functional diversity in flowering regulation [21–23]. In *Arabidopsis*, the FT/TFL1 family includes both flowering promoters, such as FT, TWIN SISTER OF FT (TSF), and MOTHER OF FT AND TFL1 (MFT), and flowering repressors like TFL1, *Arabidopsis thaliana* CENTRORADIALIS HOMOLOG (ATC), and BROTHER OF FT AND TFL1 (BFT). FT, the well-characterized ‘florigen’, is synthesized in leaves under photoperiodic induction and translocated to the shoot apical meristem (SAM) to promote flowering [24]. In contrast, TFL1 acts as a local floral inhibitor expressed in the SAM [25]. Interestingly, the functions of FT and TFL1 have diverged evolutionarily, leading to species-specific roles in regulating flowering and bolting processes and other biological development processes [26, 27]. Despite this broad understanding, the *FT*/*TFL1* gene family has not yet been fully characterized in lettuce, and its related mechanisms remain largely unclear in this crop.

In angiosperms, the floral transition and the molecular mechanisms driving the bolting–flowering process are coordinated by a sophisticated regulatory network that integrates diverse endogenous and environmental signals to ensure optimal reproductive timing [20, 28–30]. Despite extensive research in model plants, which has identified over 100 genes involved in bolting and flowering development control [28, 31–33], the key genetic factors, their precise functions, and the overarching regulatory network governing bolting and flowering in lettuce are still largely elusive. Limited numbers of genes have been identified to play a role in controlling bolting and flowering in lettuce. For instance, A DELLA protein LsRGL1 decreases the expression of *LsGA3ox* and *LsYUC4* and thus negatively regulates bolting and flowering in lettuce [34]. Auxin response factor 3 upregulates *LsCO* to mediate thermally induced transition of vegetative-to-reproductive phase in lettuce [35]. Moreover, in our previous study, we characterized two key genes (*LsBLH2* and *LsKN1*) regulating bolting time in lettuce, and demonstrated that LsBLH2 and LsKN1 transcription factors act as positive and negative regulators, respectively, forming a core module that coordinately controls bolting development through the gibberellin biosynthesis and signaling pathway [2, 3]. However, to date, only these two genes, *LsBLH2* and *LsKN1*, have been identified through forward genetics and are known to carry natural variations suitable for breeding bolting resistance. These limited genetic resources remain far from sufficient for effective bolting-resistance breeding in lettuce. Therefore, it is essential to clone additional bolting-regulatory genes harboring natural variations and to elucidate their precise regulatory mechanisms and molecular foundations governing the entire bolting process.

## Results

### Construction of a segregating population for the bolting time

To investigate the genetic basis of bolting regulation in lettuce, we generated an F_2_ population through hybridization between an early- and a late-bolting cultivar (Fig. S1A, B). This F_2_ population, consisting of 201 individuals, exhibited a continuous variation in bolting time, confirming that bolting time is a quantitative trait in lettuce (Fig. S1C). To reduce the trait complexity within the F_2_ population and to facilitate the genetic cloning of the key gene governing bolting time, F_2_ plants were self-pollinated for three to four generations, resulting in the establishment of F_4:5_ and F_5:6_ families. We assessed the bolting time, measured as the number of days after sowing (DAS) until bolting, in individuals derived from these F_4:5_ and F_5:6_ families, and an F_5:6_ family named LB356 showed either early (59–68 DAS) or late (79–91 DAS) bolting, with no intermediate phenotypes (Fig. 1A; Table S1). Within the LB356 family, comprising 196 individuals, 141 exhibited early bolting, while 55 displayed late bolting (Fig. 1A–C; Table S1). This distribution of bolting time followed a 3:1 ratio, consistent with Mendelian inheritance (χ^2^ = 0.98, *P* > 0.05), indicating that bolting time in this family is governed by a single locus and that the allele responsible for late bolting is recessive.

**Figure 1:**
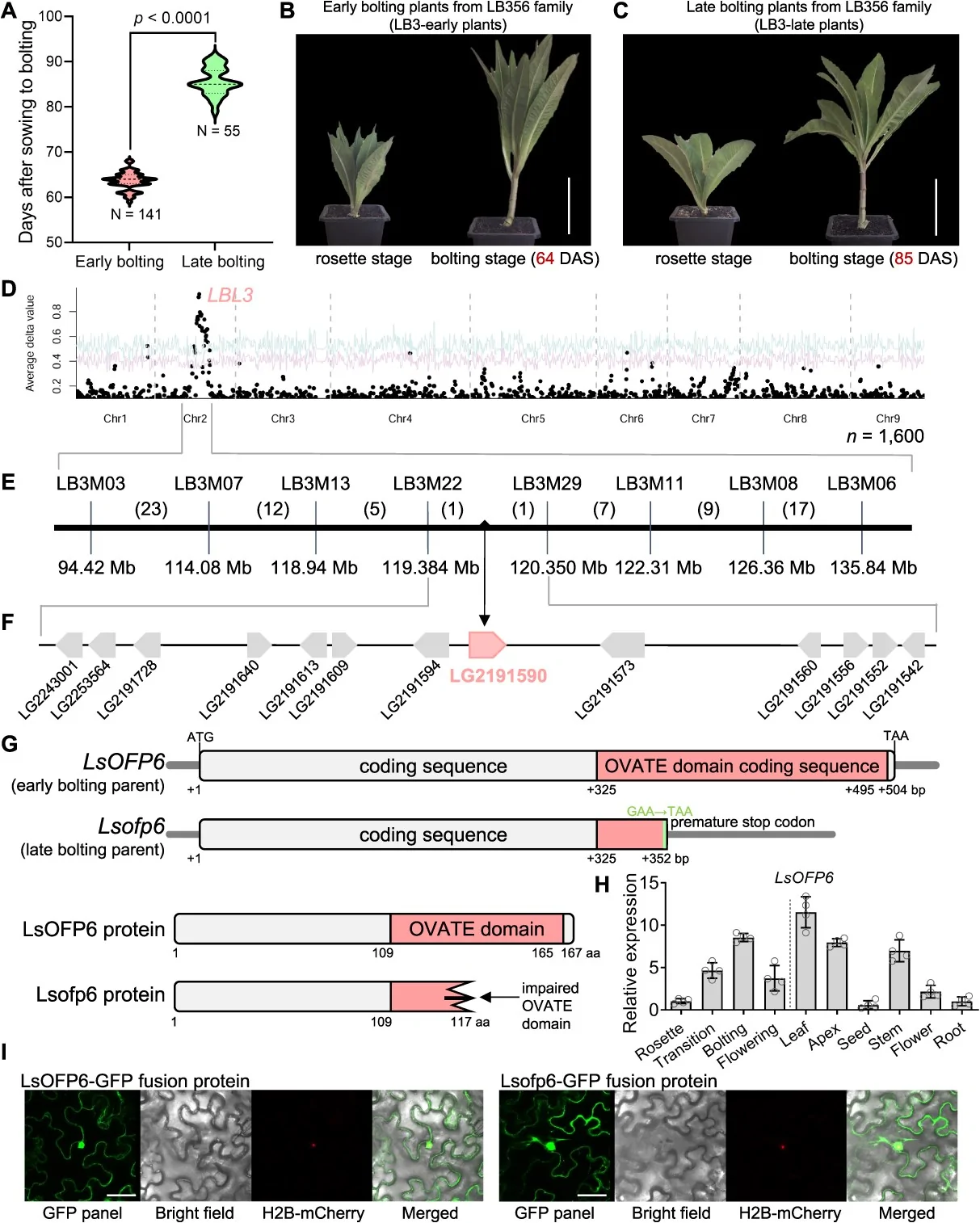
Characterization of bolting time trait in the LB356 family and map-based cloning of *LBL3* (*LsOFP6*). (A) The days after sowing (DAS) to bolting in the LB356 family. (B) The front view of the early bolting plants (LB3-early) from the LB356 family at rosette stage (left) and bolting stage (64 DAS, right). Bar = 10 cm. (C) The front view of the late bolting plants (LB3-late) from the LB356 family at rosette stage (left) and bolting stage (85 DAS, right). Bar = 10 cm. (D) BSR-seq analysis revealed a single locus on chromosome 2 that governs bolting time in the LB356 family. The magenta and green curves represent confidence intervals corresponding to *P* = 0.05 and *P* = 0.01, respectively. (E) Map-based cloning of the candidate gene. The numbers within parentheses indicate the count of recombinants identified between adjacent mapping markers. (F) Thirteen genes are present in the candidate interval. (G) Sequence variation schematic for *LsOFP6* and *Lsofp6* alleles from early and late bolting parents, respectively, and the predicted amino acid sequence encoded by the *LsOFP6* and *Lsofp6* alleles. (H) The temporal expression pattern (left) and spatial expression pattern (right) of *LsOFP6*. Data are shown as means ± SD of four biological replicates. (I) Subcellular localization of LsOFP6 and Lsofp6 in lettuce leaf cells visualized by confocal laser scanning microscopy. Observations were conducted across three biological replicates (each comprising approximately 100 nuclei). Bars = 100 μm. H2B-mCherry was used as a nuclear-localized fluorescent marker.

We applied BSR-seq to pinpoint the genetic region responsible for bolting time variation in the LB356 family. Analysis of the sequencing data identified a distinct peak on chromosome 2, which we designated as *Lettuce Bolting Locus 3* (*LBL3*) (Fig. 1D). A linked marker, LB3M07, was developed in the vicinity of *LBL3* and showed complete co-segregation with the bolting time phenotype across all 196 progenies in the LB356 family (Fig. 1E, Table S2). Consequently, we established a population in which the late/early bolting time trait is governed solely by the *LBL3*.

### Fine-mapping and cloning of the candidate gene for the bolting time trait

For the genetic fine-mapping and cloning of the *LBL3* gene, we screened 1600 progenies from the LB356 family using two flanking markers, LB3M03 (94.42 Mb) and LB3M06 (135.84 Mb), and identified 75 recombinants (Fig. 1E). By genotyping these recombinants with multiple markers derived from the candidate region, the candidate gene was narrowed down to a 966-Kb interval on chromosome 2, located between LB3M22 (119.384 Mb) and LB3M29 (120.350 Mb) (Fig. 1E). Within this candidate interval, 13 genes were predicted (Fig. 1F). Analysis of function, sequence variation, and transcript level were subsequently conducted for these 13 genes (Table S3). The result indicated that LG2191590, an *OVATE family protein 6* ortholog, has a single nucleotide polymorphism in the coding sequence between the alleles from two parents (Fig. 1G). We named LG2191590 *LsOFP6*. Compared to the *LsOFP6* allele from the early bolting parent, the allele from the late bolting parent had a guanine (G) to thymine (T) mutation at the 352nd base in the coding sequence, forming a stop codon ‘TAA’, which led to premature termination of translation of *LsOFP6* and a truncated protein with an impaired OVATE domain. We named the mutant allele *Lsofp6* (Fig. 1G). The nonsense mutation in *Lsofp6* co-segregated with the bolting time trait across all 1600 progenies of the LB356 family. Interestingly, our prior research has demonstrated that *LsOFP6* participates in regulating lettuce bolting development by interacting with LsBLH2 (BELL1-like homeobox protein) and LsKN1 (knotted-1-like homeobox family protein) [2, 3]. Thus, *LsOFP6* represents a strong candidate gene responsible for regulating bolting time in the LB356 family.

The expression levels of the *LsOFP6* gene were measured across the rosette stage, vegetative-to-reproductive transition stage, bolting stage, and flowering stage to characterize its temporal expression profile in LB3-early plants (*LsOFP6*/*LsOFP6*). The expression level of *LsOFP6* showed an increasing trend from the transition stage, peaked at the bolting stage, and subsequently declined during the flowering stage (Fig. 1H). To characterize the spatial expression of *LsOFP6*, its transcript levels were examined across leaf, shoot apex, root, seed, stem, and flower tissues. The leaf, apex, and root tissues were from the rosette stage, and the stem and flower tissues were from the bolting and flowering stage. The results indicated that *LsOFP6* was predominantly expressed in leaves, stems, and apexes while showing minimal expression in flowers, seeds, and roots (Fig. 1H). Subsequently, we investigated the subcellular localization of LsOFP6 by introducing an LsOFP6-GFP fusion gene into lettuce. The result confirmed that LsOFP6 localizes to the nucleus in lettuce cells (Fig. 1I).

### Knockout and complementation tests verified that *LsOFP6* positively controls bolting in lettuce

A specific sgRNA targeting *LsOFP6* was designed within the coding sequence of its OVATE domain to disrupt this domain in LB3-early plants using the CRISPR/Cas9 (Fig. 2A). We obtained two knockout alleles: *LsOFP6*-KO#5, which carries a 17-nucleotide deletion, and *LsOFP6*-KO#6, containing an 11-nucleotide deletion (Fig. 2A). These deletions in *LsOFP6*-KO#5 and #6 result in premature stop codons at amino acid residues 127 and 129, respectively, producing truncated LsOFP6 proteins with an impaired OVATE domain (Fig. 2B). As anticipated, the phenotype of the homozygous knockout shifted from early to late bolting (Fig. 2C and D). This confirms that LsOFP6 promotes bolting and that disruption of its OVATE domain leads to delayed bolting, as observed in LB3-late plants.

**Figure 2:**
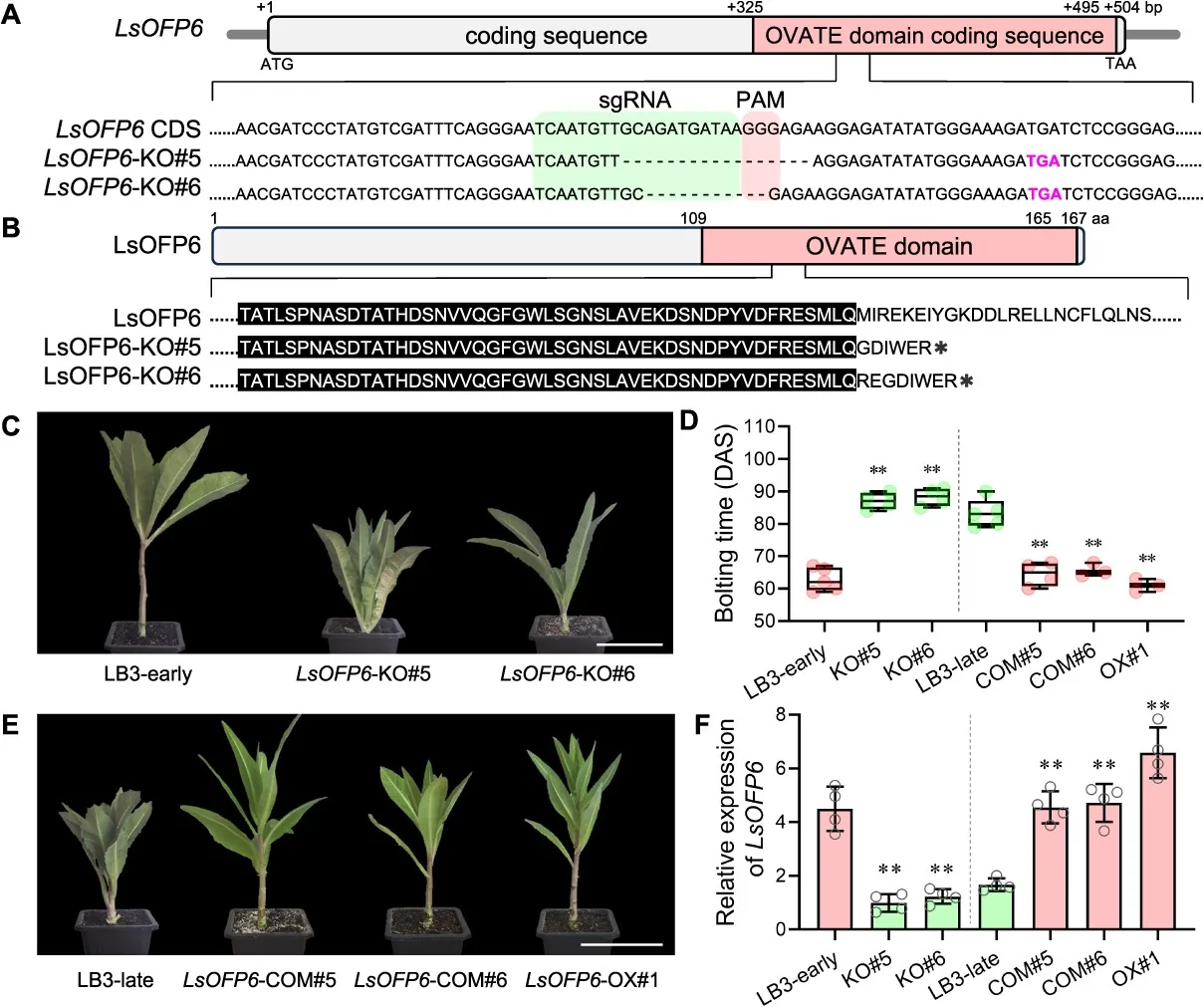
Functional validation of the role of *LsOFP6* in regulating bolting time in lettuce. (A) Sequence variation of *LsOFP6* in the knockout mutants *LsOFP6*-KO#5 and *LsOFP6*-KO#6. (B) The variation of amino acid sequences coded by the *LsOFP6*-KO#5 and *LsOFP6*-KO#6 alleles. (C) Knockout of *LsOFP6* changed the phenotype from early bolting (left) to late bolting. Bar = 10 cm. (D) The bolting time of LB3-early, *LsOFP6*-KO, LB3-late, *LsOFP6*-COM, and *LsOFP6*-OX plants. (E) *LsOFP6*-COM and *LsOFP6*-OX plants changed the phenotype from late bolting (left) to early bolting. Bar = 10 cm. (F) Transcript level of the *LsOFP6* gene in LB3-early, *LsOFP6*-KO, LB3-late, *LsOFP6*-COM, and *LsOFP6*-OX plants. Data are shown as means ± SD of four biological replicates (two-tailed Student’s *t*-test, ^**^P < 0.01).

The full *LsOFP6* allele, comprising a 2000-bp promoter, coding sequence, and a 500-bp downstream sequence, was also introduced into the LB3-late plants. Two positive transformants, designated *LsOFP6*-COM#5 and #6, were obtained (Fig. 2E). Both complementary lines exhibited earlier bolting compared to the recipient LB3-late plants (Fig. 2D and E). We also overexpressed *LsOFP6* in the LB3-late plants, and the *LsOFP6*-OX#1 line also exhibited earlier bolting compared to the recipient (Fig. 2D–F). These results further support the role of *LsOFP6* in promoting the transition from vegetative growth to reproductive development in lettuce.

### LsOFP6-mediated bolting regulation is independent of GA in the *Lsblh2 Lskn1* background

Based on our previous findings that LsOFP6 regulates lettuce bolting by interacting with LsBLH2 and LsKN1 to modulate gibberellin (GA) biosynthesis, we genotyped the LB356 family for these two genes. The results revealed that the LB356 family fixed a homozygous loss-of-function allele for *LsBLH2* (*Lsblh2*) and a homozygous inactive allele for *LsKN1* (*Lskn1*). This genetic analysis indicates that LsOFP6 regulates bolting in an LsBLH2- and LsKN1-independent manner in the LB356 family with genetic background *Lsblh2 Lskn1*.

Furthermore, we quantified GA levels at multiple developmental stages in both LB3-early (*LsOFP6*) and LB3-late (*Lsofp6*) plants. No significant difference in GA content was detected between the LB3-early and LB3-late plants at any stage (Fig. S2A–H). Consistent with these results, the expression of key GA biosynthesis/catabolism genes (*LsGA20ox1*, *LsGA2ox8*, and *LsGA2ox4*) also showed no significant variation across the stages assayed (Fig. S2I–T). These results collectively demonstrated that LsOFP6 regulates bolting through a GA-independent pathway in the LB356 family with genetic background *Lsblh2 Lskn1*.

### *LsOFP6* physically interacts with *LsCOL3*

We then explored whether LsOFP6 regulates bolting development through interacting with other proteins in lettuce. To identify potential interacting partners, a yeast two-hybrid (Y2H) screening was conducted with a lettuce cDNA library. This screen identified 20 candidate proteins (Table S4), including a CONSTANS-like (COL) protein (gene identifier: LG5518041), which belongs to the known flowering time regulator family, CCT (CONSTANS, CO-like, and TOC1) family. The lettuce genome contains 14 COL genes, while *Arabidopsis* has 17 [17]. A phylogenetic tree was constructed for all 31 COL proteins from lettuce and *Arabidopsis*, using BBX20 (AT4G39070) as an outgroup for comparison (Figs 3A and S3). According to the phylogenetic tree, LG2255123 is orthologous to AtCO (AT5G15840), AtCOL1 (AT5G15850), and AtCOL2 (AT3G02380); LG5518041, LG8728102, and LG5487786 are orthologous to AtCOL3/4 (AT2G24790, AT5G24930); LG3290364 and LG7632711 are orthologous to AtCOL6/16 (AT1G68520, AT1G25440); LG9775456, LG5497722, and LG7619064 are orthologous to AtCOL9/10 (AT3G07650, AT5G48250); LG4403319 is orthologous to AtCOL11/12 (AT4G15250, AT3G21880); LG9797271 is orthologous to AtCOL13 (AT2G47890); and LG8721313 is orthologous to AtCOL14/15 (AT2G33500, AT1G28050) (Figs 3A and S3). For convenience, we hereafter refer to LG5518041 as LsCOL3.

**Figure 3:**
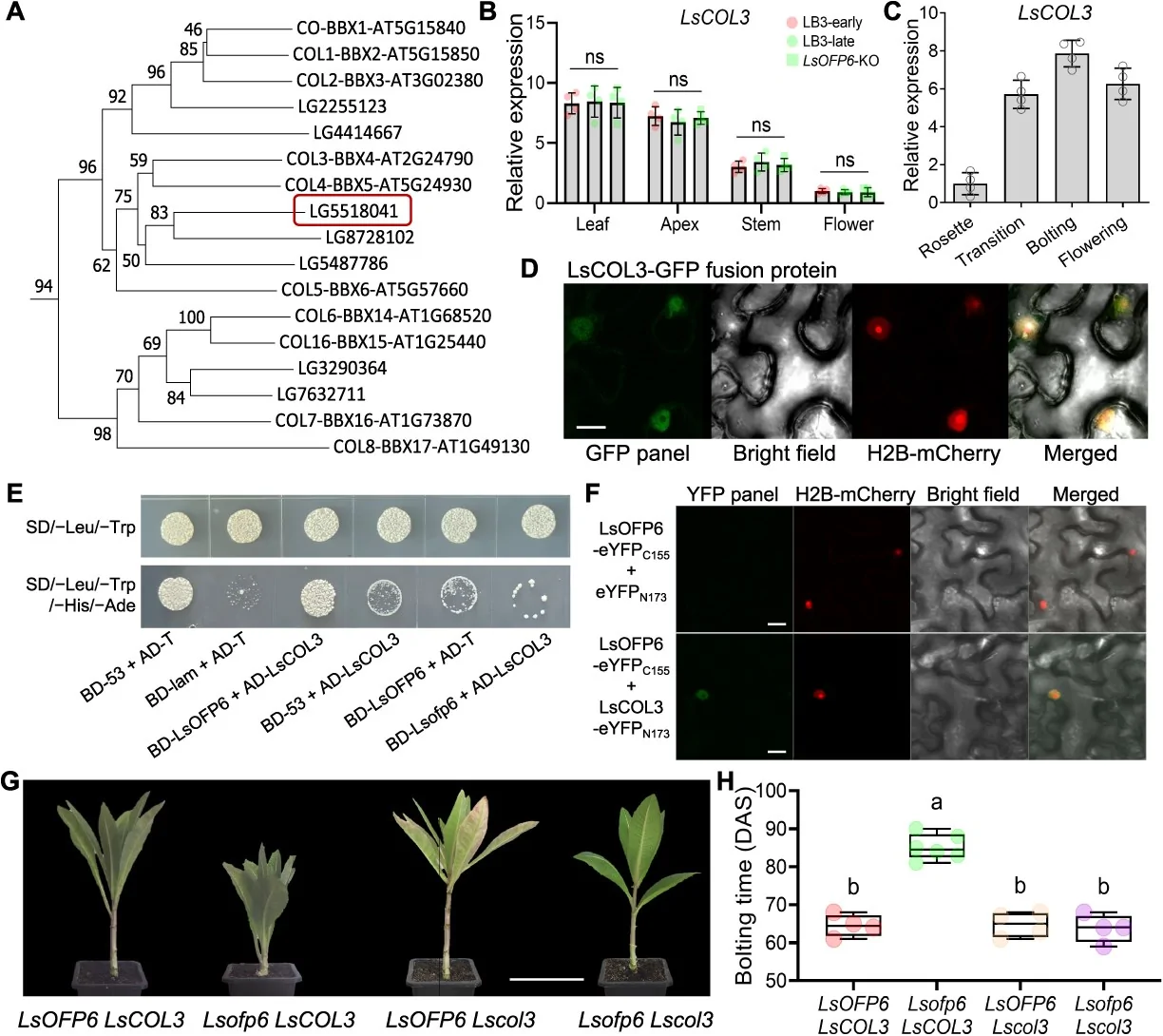
LsOFP6 and LsCOL3 physically interact and function synergistically to control bolting development. (A) A neighbor-joining phylogenetic tree was constructed by using the amino acid sequences of CONSTANS-LIKE proteins (COLs) from lettuce and *Arabidopsis*. LsCOL3 (LG5518041) is indicated with a red rounded rectangle. Numbers on nodes refer to the bootstrap value. The complete tree is shown in Fig. S3. (B) The relative expression of the *LsCOL3* gene in different tissues from LB3-early, LB3-late, and *LsOFP6*-KO plants. Data are shown as means ± SD of four biological replicates (two-tailed Student’s *t*-test, ns for not significant). (C) The temporal expression pattern of *LsCOL3* gene. Data are shown as means ± SD of four biological replicates. (D) Subcellular localization of LsCOL3 in *N. benthamiana* leaf cells visualized by confocal laser scanning microscopy. Scale bar = 25 μm. H2B-mCherry was used as a nuclear-localized fluorescent marker. (E) Y2H assay shows the LsOFP6–LsCOL3 interaction. (F) BiFC assay shows LsOFP6–LsCOL3 interaction. Scale bars = 25 μm. H2B-mCherry was used as a nuclear-localized fluorescent marker. eYFP_N173_ represents the N-terminal 1–173 amino acids of eYFP. eYFP_C155_ represents the C-terminal 156–239 amino acids of eYFP. (G, H) The front view (G) and the bolting time phenotype (H) of *LsOFP6 LsCOL3*, *Lsofp6 LsCOL3*, *LsOFP6 Lscol3*, and *Lsofp6 Lscol3* plants. Scale bar = 10 cm. Different letters indicate significant differences (ANOVA followed by Tukey’s multiple comparison test, *P* < 0.05).

We next examined the spatial expression pattern of the *LsCOL3* gene by measuring the transcript levels of the *LsCOL3* gene in different tissues from LB3-early, LB3-late, and *LsOFP6*-KO plants. The results revealed no significant difference in *LsCOL3* expression among the LB3-early (*LsOFP6*), LB3-late (*Lsofp6*), and *LsOFP6*-KO plants, indicating that LsOFP6 does not affect the expression of *LsCOL3* (Fig. 3B). *LsCOL3* was predominantly expressed in leaves, apexes, and stems, with minimal expression observed in other tissues, indicating that *LsCOL3* has a similar expression pattern to *LsOFP6* (Figs 3B and 1H). We also examined the temporal expression pattern of *LsCOL3*, and the result showed that LsCOL3 has a similar temporal expression pattern to *LsOFP6* (Fig. 3C and 1H). Then, we identified the subcellular localization of LsCOL3 through transiently expressing the fusion gene *LsCOL3*-*GFP* in *Nicotiana benthamiana* leaves using *Agrobacterium*-mediated transformation. The result showed that LsCOL3 is co-localized with LsOFP6 at the nuclei (Fig. 3D and 1I).

We used the Y2H system to further confirm the interaction between LsOFP6 and LsCOL3 (Fig. 3E). Then, we applied the BiFC assay by co-expressing 35S:LsOFP6-eYFP_C155_ and 35S:LsCOL3-eYFP_N173_ in *N*. *benthamiana* leaf cells, and the result confirmed the LsOFP6–LsCOL3 protein interaction *in vivo* (Fig. 3F). The results from both Y2H and BiFC assays consistently demonstrated a physical interaction between LsOFP6 and LsCOL3, suggesting that LsOFP6 may regulate lettuce bolting through its association with LsCOL3.

### *LsOFP6* and *LsCOL3* synergistically regulate bolting development

We therefore investigated the role of LsCOL3 in regulating bolting development, as well as the genetic relationship between *LsOFP6* and *LsCOL3* in this process. We first knocked out *LsCOL3* in LB3-early (*LsOFP6 LsCOL3*) and LB3-late (*Lsofp6 LsCOL3*) plants to create *LsOFP6 Lscol3* and *Lsofp6 Lscol3* genotype plants, respectively (Fig. 3G). Interestingly, the results showed that the bolting time has no significant difference between *LsOFP6 Lscol3* and *Lsofp6 Lscol3* (both show early bolting), but the bolting time in *Lsofp6 LsCOL3* plants is significantly later than that in *LsOFP6 LsCOL3* plants (Fig. 3G and H). Therefore, *LsOFP6* positively regulates bolting in a *LsCOL3*-dependent manner. In addition, the bolting time has no significant difference between *LsOFP6 LsCOL3* and *LsOFP6 Lscol3* (both show early bolting), while the bolting time in *Lsofp6 LsCOL3* plants is significantly later than that in *Lsofp6 Lscol3* plants (Fig. 3G and H). Therefore, *LsCOL3* negatively regulates bolting and relies on *LsOFP6* in a dominant epistatic manner.

The above genetic analysis results indicate that *LsOFP6* positively regulates bolting in a *LsCOL3*-dependent manner (i.e. *LsOFP6* relies on *LsCOL3* in a recessive epistatic manner), while *LsCOL3* negatively regulates bolting, but this effect requires dysfunctional *LsOFP6* (i.e. *LsCOL3* relies on *LsOFP6* in a dominant epistatic manner).

### LsCOL3 downregulates *LsTSF1* and upregulates *LsTFL1* genes to repress bolting development

To explore the molecular mechanisms through which LsCOL3 regulates bolting development, DNA affinity purification sequencing (DAP-seq) was performed to identify genomic regions directly bound by the LsCOL3 transcription factor. Preliminary data analysis revealed LG2192922 and LG2241145 as candidate genes potentially regulated by the LsCOL3 transcription factor in a direct manner. Both LG2192922 and LG2241145 encode *FT*/*TFL1* family genes (Fig. 4A). Further analysis showed that both the lettuce and the *Arabidopsis* genomes encode six *FT*/*TFL1* family genes. A phylogenetic tree was constructed by using the 12 FT/TFL1 proteins encoded by the lettuce and *Arabidopsis* genomes, with the STEPMOTHER OF FT AND TFL1 (SMFT, AT5G01300) as the outgroup (Fig. 4A). According to the phylogenetic tree, LG2192922 is orthologous to FT (AT1G65480) and TSF (AT4G20370); LG2241145 and LG8762796 are orthologous to TFL1 (AT5G03840) (Fig. 4A). LG2192922 exhibits 63.8% amino acid identity to FT and 73.7% to TSF (Figs S4 and S5). We refer to LG2192922 as LsTSF1 and LG2241145 as LsTFL1.

**Figure 4:**
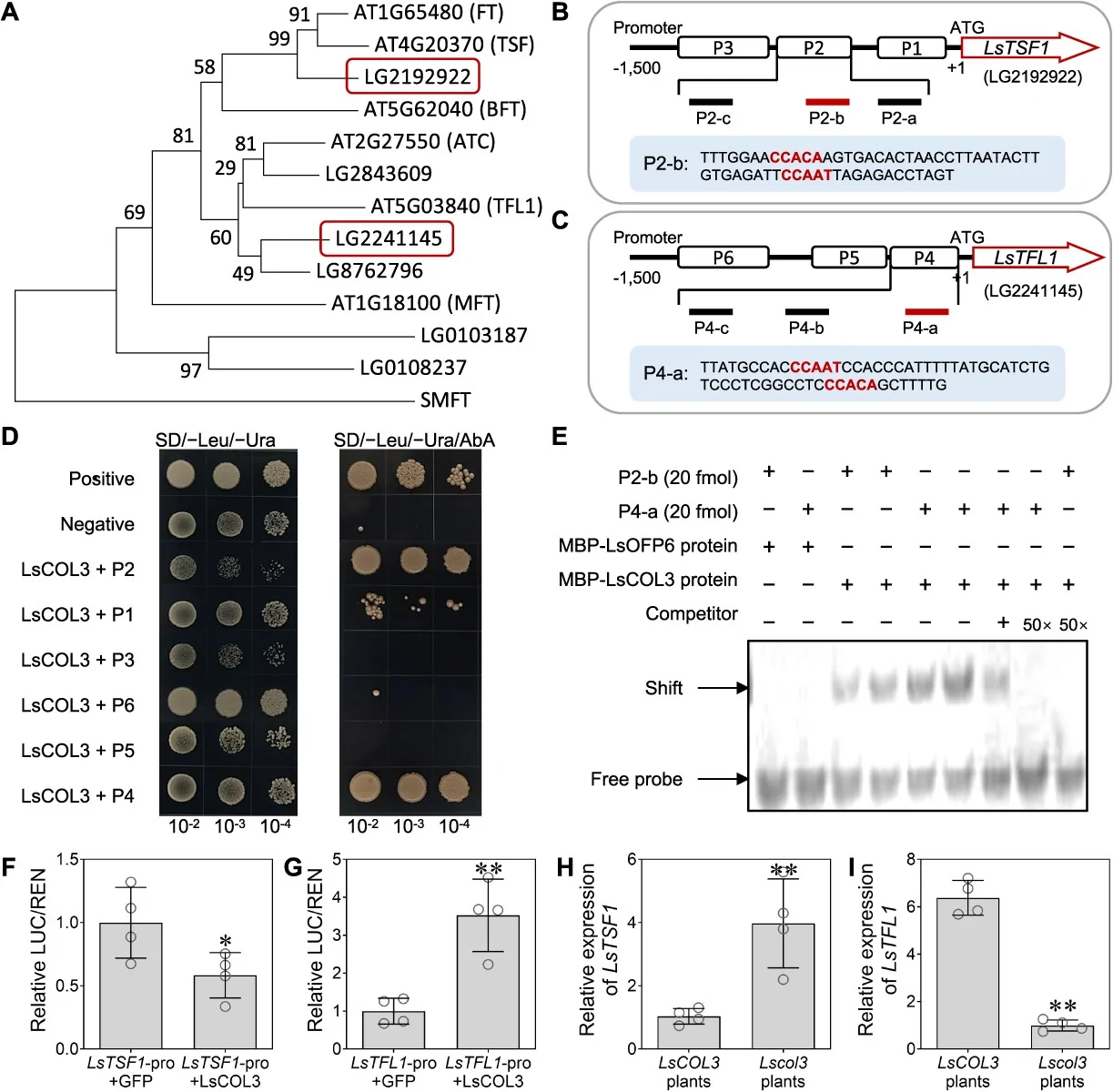
LsCOL3 regulates bolting by transcriptional control of *LsTSF1* and *LsTFL1* in lettuce. (A) Neighbor-joining phylogenetic tree using amino acid sequences encoded by *FT*/*TFL1* family genes from lettuce and *Arabidopsis*. LsTSF1 (LG2192922) and LsTFL1 (LG2241145) are indicated with red rounded rectangles. Numbers on the nodes indicate bootstrap values. (B and C) Preliminary analysis of DAP-seq revealed *LsTSF1* (B) and *LsTFL1* (C) as potential direct targets of the LsCOL3 transcription factor. P1, P2, P3, P4, P5, and P6 fragments were used as baits in the Y1H. P2-a, P2-b, P2-c, P4-a, P4-b, and P4-c fragments were used as probes in the EMSA assay. (D) Y1H analysis demonstrates that LsCOL3 binds directly to the P2 and P4 fragments. (E) EMSA analysis demonstrates that LsCOL3 binds directly to the P2-b and P4-a fragments. (F and G) Dual-luciferase assay suggests that LsCOL3 downregulates *LsTSF1* (F) and upregulates *LsTFL1* (G). Data are shown as means ± SD of four biological replicates (two-tailed Student’s *t*-test, ^*^P < 0.05, ^**^P < 0.01). (H and I) The relative expression of *LsTSF1* (H) and *LsTFL1* (I) genes in the *LsCOL3* and *Lscol3* plants. Data are shown as means ± SD of four biological replicates (two-tailed Student’s *t*-test, ^**^P < 0.01).

Notably, COLs act as transcriptional regulators of *FT*/*TFL1* family genes by binding to their promoters, as demonstrated in previous studies [36–38]. Thus, we propose that LsCOL3 interacts with the promoters of *LsTSF1* and *LsTFL1*, modulating their expression to regulate bolting in lettuce. To evaluate this, yeast one-hybrid (Y1H) assay and electrophoretic mobility shift assay (EMSA) were conducted. Both assays demonstrated that LsCOL3 binds to the promoter regions of *LsTSF1* and *LsTFL1* genes (Fig. 4B–E). Indeed, the sequences of P2-b and P4-a probes from the *LsTSF1* and *LsTFL1* promoter, respectively, contain the ‘CCACA’ and ‘CCAAT’ motifs, which are potential binding motifs of COLs (Fig. 4B and C) [36, 37].

We further employed a dual-luciferase assay to examine how LsCOL3 influences the regulation of *LsTSF1* and *LsTFL1* (Fig. S6). The data revealed that the LUC enzyme activity in the combination ‘35S:*LsCOL3* + *LsTSF1*-Pro:*LUC*’ was significantly reduced compared to that in the control ‘35S:*GFP* + *LsTSF1*-Pro:*LUC*’ (Fig. 4F). Conversely, the LUC enzyme activity in the combination ‘35S:*LsCOL3* + *LsTFL1*-Pro:*LUC*’ was significantly increased compared to that in the control ‘35S:*GFP* + *LsTFL1*-Pro:*LUC*’ (Fig. 4G). Furthermore, in the *Lsofp6* genotype background, the transcript level of *LsTSF1* in *LsCOL3* plants was significantly lower than that in *Lscol3* plants, and the transcript level of *LsTFL1* in *LsCOL3* plants was significantly higher than that in *Lscol3* plants (Fig. 4H, I). These data indicated that LsCOL3 directly binds the promoter of *LsTSF1* and *LsTFL1* and downregulates and upregulates their expression, respectively, to delay bolting in lettuce.

### LsOFP6 attenuates the effects of LsCOL3 on *LsTSF1* and *LsTFL1*

To investigate whether LsOFP6 influences the LsCOL3-mediated regulation of *LsTSF1* and *LsTFL1* expression, and to clarify the molecular mechanism through which the LsOFP6–LsCOL3 module controls bolting development in lettuce, we first measured the transcript levels of *LsTSF1* in plants with different genotypic combinations: *LsOFP6 LsCOL3*, *Lsofp6 LsCOL3*, *LsOFP6 Lscol3*, and *Lsofp6 Lscol3*. The results showed that the transcript level of *LsTSF1* in the *Lsofp6 LsCOL3* genotype was significantly lower than that in other genotypes (Fig. 5A). Interestingly, no significant difference in the transcript level of *LsTSF1* was observed among *LsOFP6 LsCOL3*, *LsOFP6 Lscol3*, and *Lsofp6 Lscol3* plants (Fig. 5A). We also performed dual-luciferase assays to further investigate the combinatorial effects of LsOFP6 and LsCOL3 on the transcriptional regulation of *LsTSF1* (Figs 5B and S6). The *LUC* gene is driven by the *LsTSF1* promoter, and the result revealed that the LUC activity in group #2 (co-expressing *Lsofp6* and *LsCOL3*) is significantly lower than that in other groups (Fig. 5B). Additionally, there was no significant difference in LUC activity observed among groups #1, #3, and #4 (Fig. 5B).

**Figure 5:**
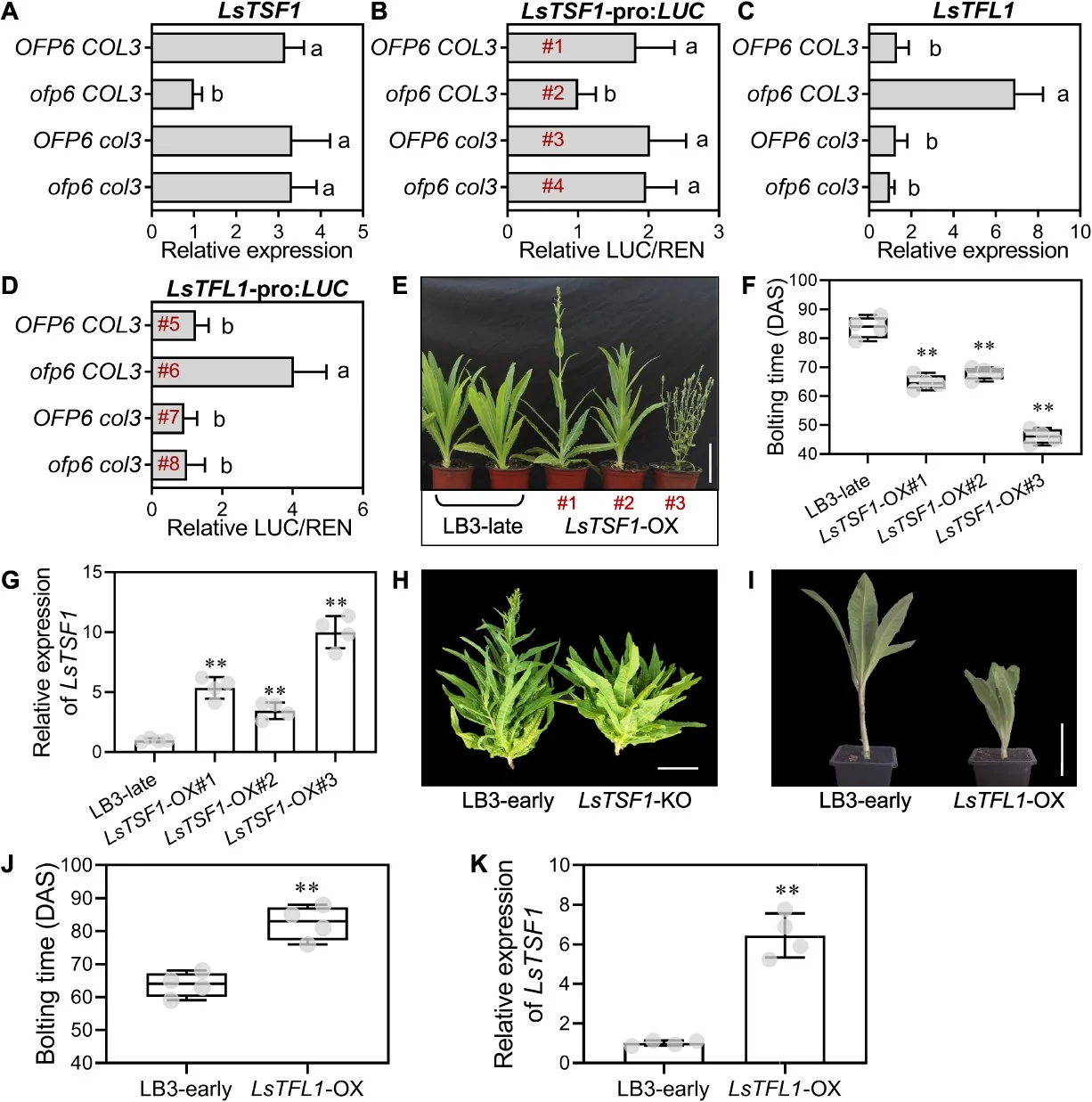
LsOFP6 compromised the effects of LsCOL3 on downregulating *LsTSF1* and upregulating *LsTFL1*. (A) Relative expression of *LsTSF1* in *LsOFP6 LsCOL3*, *Lsofp6 LsCOL3*, *LsOFP6 Lscol3*, and *Lsofp6 Lscol3* plants. Data are shown as means ± SD of four biological replicates (Tukey’s multiple comparison test, *P* < 0.05). (B) The LUC activity observed when the *LUC* gene was driven by the *LsTSF1* promoter and co-expressed with different alleles of *LsOFP6*/*Lsofp6* and *LsBLH2*/*Lsblh2*. Data are shown as means ± SD of four biological replicates (Tukey’s multiple comparison test, *P* < 0.05). (C) Relative expression of *LsTFL1* in *LsOFP6 LsCOL3*, *Lsofp6 LsCOL3*, *LsOFP6 Lscol3*, and *Lsofp6 Lscol3* plants. Data are shown as means ± SD of four biological replicates (Tukey’s multiple comparison test, *P* < 0.05). (D) The LUC activity observed when the *LUC* gene was driven by the *LsTFL1* promoter and co-expressed with different alleles of *LsOFP6*/*Lsofp6* and *LsBLH2*/*Lsblh2*. Data are shown as means ± SD of four biological replicates (Tukey’s multiple comparison test, *P* < 0.05). (E–G) The front view (E), the bolting time phenotype (F), and the expression of the *LsTSF1* (G) in the LB3-late and *LsTSF1*-OX plants. Scale bar = 10 cm. Significant differences were determined by *t*-test (^**^P < 0.01). (H) The front view of LB3-early and *LsTSF1*-KO plants. Scale bar = 10 cm. (I–K) The front view (I), the bolting time phenotype (J), and the expression of the *LsTFL1* gene (K) in the LB3-late and *LsTFL1*-OX plants. Scale bar = 10 cm. *t*-test (^**^P < 0.01).

We also quantified the transcript level of *LsTFL1* in the four genotypes. The results revealed that the transcript level of *LsTFL1* in *Lsofp6 LsCOL3* genotype was significantly higher than that in other genotypes, and no significant difference in the transcript level of *LsTFL1* was observed among *LsOFP6 LsCOL3*, *LsOFP6 Lscol3*, and *Lsofp6 Lscol3* plants (Fig. 5C). The dual-luciferase assays showed that the LUC activity in group #6 is significantly higher than that in other groups, and the LUC activity has no obvious changes among groups #5, #7, and #8 (Fig. 5D).

The above results indicated that *LsOFP6* compromises the effects of LsCOL3 on downregulating *LsTSF1* and upregulating *LsTFL1*; the transcriptional regulation of LsCOL3 on *LsTSF1* and *LsTFL1* requires the nonfunctional *Lsofp6*; *LsOFP6* regulates the expression of *LsTSF1* and *LsTFL1* genes in a *LsCOL3*-dependent manner. Therefore, we hypothesized that the loss of function of *Lsofp6* downregulates *LsTSF1* and upregulates *LsTFL1* to delay the transition of the vegetative growth to reproductive growth in lettuce.

To test this hypothesis, we first overexpressed *LsTSF1* in the LB3-late plants (genotype: *Lsofp6 LsCOL3*) (Fig. 5E). All three *LsTSF1*-OX plants showed early bolting compared with the recipients LB3-late plants (Fig. 5E–G). Then, the specific sgRNA for *LsTSF1* was designed in the coding sequence to knock out *LsTSF1* in LB3-early plants (genotype: *LsOFP6 LsCOL3*) using the CRISPR/Cas9 system. The *LsTSF1*-KO plants lost the ability to transition from vegetative to reproductive growth (Fig. 5H). The *LsTFL1* gene was also overexpressed in the LB3-early plants (Fig. 5I). The *LsTFL1*-OX plants showed late bolting compared with the recipients (Fig. 5I–K).

Therefore, *LsOFP6* compromises the effects of LsCOL3 on downregulating *LsTSF1* and upregulating *LsTFL1*, leading to activated *LsTSF1* and suppressed *LsTFL1*, and consequently, early bolting (Fig. 6). The late bolting phenotype in *Lsofp6* mutant results from the low expression of *LsTSF1* and high expression of *LsTFL1*, which are suppressed and activated by LsCOL3, respectively (Fig. 6).

**Figure 6:**
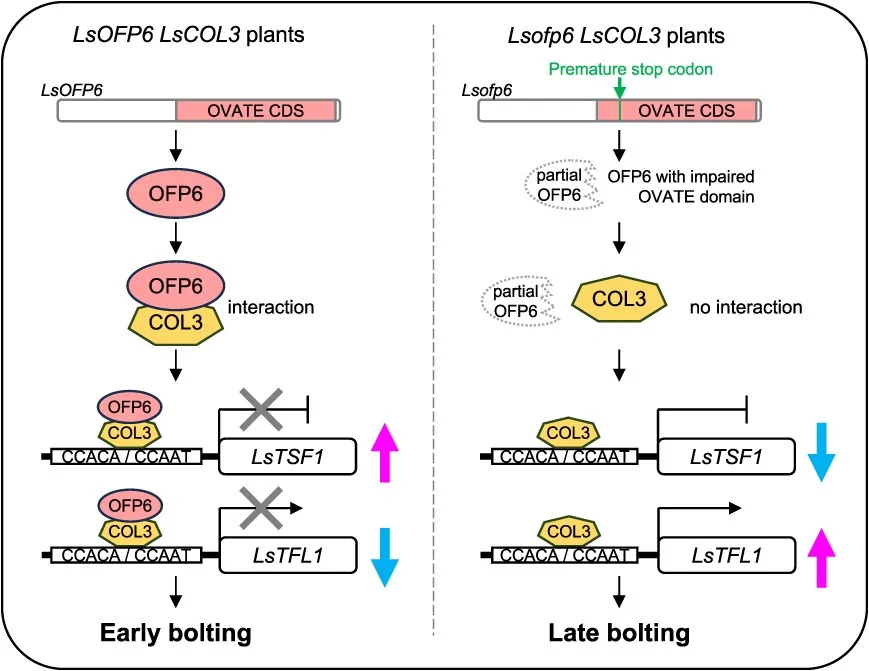
LsOFP6 and LsCOL3 synergistically regulate bolting time by transcriptional control of *FT*/*TFL1* family genes in lettuce.

## Discussion

### LsOFP6 regulates bolting development by modulating multiple biological pathways

The regulation of bolting time is a pivotal agronomic determinant in lettuce production, as premature bolting compromises both nutritional quality and textural attributes, thereby undermining market competitiveness. However, the genetic architecture and molecular mechanisms governing bolting time in lettuce remain insufficiently elucidated, which hampers progress in precision breeding aimed at this trait. Currently available genetic resources for bolting-related genes are limited, and regulatory pathways may vary across cultivars, collectively posing significant challenges to genetic improvement.

In our study, an *OVATE protein family* gene, *LsOFP6*, was genetically cloned (Fig. 1D–G). Although members of the OFP family are involved in transcriptional regulation, they do not possess typical DNA-binding domains. Instead, they act through protein–protein interactions with transcription factors to mediate specific biological processes [7]. Indeed, our EMSA assay showed that LsOFP6 protein alone did not cause any mobility shift (Fig. 4E), confirming that LsOFP6 lacks intrinsic DNA-binding activity and does not directly target these promoters. OFPs exhibit a close functional association with TALEs, including BLHs and KNOXs. The OFP–TALE complexes play a critical role in plant developmental processes [12]. Indeed, our previous work established that LsOFP6 enhances the effects of LsBLH2 on upregulating *LsGA20ox1* and downregulating *LsGA2ox8*, and LsOFP6 attenuates the effects of LsKN1 on upregulating *LsRGA1* and downregulating *LsGA20ox1* [2, 3].

Interestingly, the mapping population (LB356 family) fixed a homozygous loss-of-function allele for *LsBLH2* (*Lsblh2*) and a homozygous inactive allele for *LsKN1* (*Lskn1*), indicating that LsOFP6 regulates bolting in an LsBLH2- and LsKN1-independent manner in the LB356 family with genetic background *Lsblh2 Lskn1*. Further analysis showed that LsOFP6 physically interacts with LsCOL3 to control the expression of *FT*/*TFL1* family genes, and LsOFP6 regulates bolting development in a LsCOL3-dependent manner in the LB356 family. LsOFP6 compromised the effects of LsCOL3 on downregulating the expression of *LsTSF1* and upregulating the expression of *LsTFL1*, resulting in early bolting (Fig. 6). In the late bolting parent, the *Lsofp6* allele has a nonsense mutation and encodes a truncated protein with an impaired OVATE domain (Fig. 1G). Compared to *LsOFP6* plants, *Lsofp6* plants exhibit lower *LsTSF1* expression and higher *LsTFL1* expression, which collectively contribute to delayed bolting.

These results indicated that LsOFP6 regulates bolting development by modulating multiple biological pathways and regulating different downstream genes, such as *LsGA20ox1*, *LsGA2ox8*, *LsTSF1*, and *LsTFL1*. Interestingly, the bolting time did not differ between *LsOFP6* and *Lsofp6* plants in the *Lscol3 Lsblh2 Lskn1* triple mutant background (Fig. 3G–H), suggesting that LsOFP6 regulates bolting mainly through interacting with LsCOL3, LsBLH2, and LsKN1. Building on our previous genetic analyses [2, 3], we propose a model where the lettuce bolting phenotype is determined by the specific protein partners available to LsOFP6. In the *Lsblh2 Lskn1* genotype background, where LsBLH2 and LsKN1 are nonfunctional, the LsOFP6–LsCOL3 module appears to be the predominant pathway regulating bolting, independently of GA signaling. Conversely, in genetic backgrounds where LsBLH2 and/or LsKN1 are functional, LsOFP6 may form complexes that primarily modulate the GA pathway, which could play a more dominant role in controlling bolting time in those contexts. This partitioning model suggests that LsOFP6 acts as a molecular hub, integrating signals from different pathways to fine-tune the bolting time in a genotype-dependent manner.

### *FT*/*TFL1* family genes play important roles in the transition of the vegetative-to-reproductive phase in horticultural crops

Bolting and flowering constitute a pivotal developmental transition in horticultural crops, involving a series of biologically coordinated processes such as floral induction, bud differentiation, and anthesis [28]. From an applied perspective, this phase not only determines reproductive success but also fundamentally influences yield formation, quality traits, and market supply scheduling. A deeper understanding of how environmental factors, including photoperiod, temperature, and abiotic stress, regulate the floral transition through interconnected signaling networks holds substantial theoretical and practical importance [29]. Such insights can identify key targets for precise flowering manipulation, enabling the development of cultivars tailored to diverse ecological regions and cultivation systems through molecular breeding. Moreover, by integrating environmental simulation and growth regulation technologies, season-independent production can be achieved, thereby enhancing product marketability, reducing energy and production costs, and ensuring a stable year-round supply of fresh horticultural produce, offering new frameworks for the sustainable optimization of horticultural production systems [39, 40].

In horticultural crops, genes within the *FT*/*TFL1* family exhibit conserved roles in regulating the floral transition. For instance, both in *Chrysanthemum seticuspe* (Asteraceae, the largest flowering plant family) and *Phalaenopsis* (Orchidaceae, the second largest family of flowering plants), three *FT*-*like* genes have been identified as flowering inducers, and their heterologous overexpression causes an early flowering phenotype, while several *TFL1* homologs act as flowering inhibitors [41–43]. In tomato (*Solanum lycopersicum*), the timing of flowering is co-regulated by the floral activator SINGLE FLOWER TRUSS (SFT) and the repressor SELF PRUNING (SP) [44]. Functioning as an FT homolog, SFT is produced in mature leaves and acts systemically to stimulate flowering. Conversely, SP, an ortholog of TFL1, accumulates in young leaves and shoots where it functions to inhibit the floral transition [45].

In this study, six *FT*/*TFL1* gene family members were identified in the lettuce genome, including an FT/TSF homolog, an ATC homolog, two TFL1 homologs, and other members (Fig. 4A). We demonstrated that *FT*/*TFL1* family genes function as key regulators of bolting development in lettuce (Fig. 5E–K). Indeed, overexpressing *LsTSF1* in LB3-late plants changes the late bolting to early bolting, while overexpressing *LsTFL1* in LB3-early plants changes the early bolting to late bolting (Fig. 5E–K). Notably, *LsTSF1* knock-out plants lost the ability of transition of vegetative-to-reproductive phase (Fig. 5H). Therefore, *FT*/*TFL1* gene family members play important and different roles in lettuce bolting development. In addition, our study identifies LsOFP6–LsCOL3 as a novel protein-level interaction module that acts as a critical switch upstream of these *FT*/*TFL1* gene family members, adding a significant new layer to the understanding of how bolting time is precisely controlled in lettuce. This represents a conceptual advance beyond the canonical transcriptional activation of FT by CO.

### LsOFP6 and LsCOL3 synergistically regulate bolting development

We clarified the genetic relationship between *LsOFP6* and *LsCOL3*, and demonstrated that the LsOFP6–LsCOL3 module regulates bolting by transcriptional control of *LsTSF1* or *LsTFL1* genes in lettuce (Fig. 6). Interestingly, no significant difference in bolting time was observed between *LsOFP6 Lscol3* and *Lsofp6 Lscol3* plants (Fig. 3G and H). Similarly, the expression levels of *LsTSF1* and *LsTFL1* did not exhibit notable variation between the two genotypes (Fig. 5A and C). Moreover, the expression of the *LUC* reporter gene, driven by either the *LsTSF1* or *LsTFL1* promoter, did not differ significantly between the group co-expressing *LsOFP6* and *Lscol3* and the group co-expressing *Lsofp6* and *Lscol3* (Fig. 5B and D). These data suggest that *Lscol3* is recessive epistatic to *LsOFP6*, and *LsOFP6* regulates bolting time in a *LsCOL3*-dependent manner.

Furthermore, bolting time remained unchanged between *LsOFP6 LsCOL3* and *LsOFP6 Lscol3* plants, whereas *Lsofp6 LsCOL3* plants exhibited significantly later bolting compared to *Lsofp6 Lscol3* plants (Fig. 3G and H). Similarly, the expression of *LsTSF1*/*LsTFL1* showed no significant difference between *LsOFP6 LsCOL3* and *LsOFP6 Lscol3* plants, but was markedly different between *Lsofp6 LsCOL3* and *Lsofp6 Lscol3* plants (Fig. 5A and C). These results demonstrate that the regulation of *LsCOL3* on bolting and the expression of *LsTSF1*/*LsTFL1* require dysfunctional *LsOFP6* (i.e. *LsCOL3* relies on *LsOFP6* in a dominant epistatic manner).

We further examined the transcript levels of *LsOFP6* and *LsCOL3* across a lettuce natural population, comprising wild lettuce accessions (*Lactuca serriola*, *Lactuca saligna*, and *Lactuca virosa*) and 198 cultivated varieties (Fig. S7A; Table S5). The results revealed a negative correlation between the expression levels of *LsOFP6* and *LsCOL3* (*P* = 7.6 × 10^−6^), supporting our conclusion that *LsOFP6* and *LsCOL3* exert antagonistic effects on the bolting development regulation in lettuce (Fig. S7B). In addition, the expression of *LsCOL3* varies among different lettuce cultivars, suggesting that its expression may be a key factor in cultivar domestication (Fig. S7A). Indeed, different cultivars require distinct bolting times. For example, stem lettuce (a stem-consuming type) needs earlier bolting, while heading lettuce requires later bolting to accumulate more biomass.

In addition, some members of the COL family act as positive regulators of flowering while others function as repressors. This divergence in function does not appear to be strictly correlated with subfamily classification, as even closely related COL members can exhibit opposite regulatory roles [19]. Such functional complexity within the COL family underscores how plants utilize a limited genetic toolkit to achieve precise developmental outcomes through elaborated regulatory networks [14]. By integrating endogenous and exogenous stimuli into differential transcriptional programs, COLs collectively ensure that flowering occurs at the optimal time, thereby enhancing reproductive success [15]. From a horticultural perspective, this regulatory flexibility offers promising avenues for crop improvement [39].

In this study, we elucidate the molecular mechanism whereby the LsOFP6–LsCOL3 transcriptional module coordinately regulates *FT*/*TFL1* family genes to control bolting time in lettuce (Fig. 6). The role of LsOFP6 in regulating bolting time was not anticipated from studies in *Arabidopsis* and other model crops, underscoring the importance of forward genetics in diverse species. From a practical breeding perspective, our findings strongly support that manipulating the LsOFP6–LsCOL3 module or its downstream targets is a viable strategy for breeding next-generation lettuce cultivars with delayed bolting and improved bolt tolerance.

## Materials and methods

### BSR-seq

Bulked segregant analysis integrated with RNA sequencing (BSR-seq) was employed to identify genomic regions associated with bolting time in lettuce [46, 47]. For this purpose, two DNA bulks were constructed: an ‘early bolting’ pool and a ‘late bolting’ pool, each formed by combining equal quantities of tissue samples from 20 individuals exhibiting distinct bolting phenotypes. Total RNA was isolated from each pooled sample. Sequencing was carried out on the DNBSEQ-T7 platform (Smartgenomics, China), yielding ~6 Gb of high-quality reads per pool. Bioinformatics processing and data interpretation followed the methodology outlined [48]. The primers utilized in genetic mapping are listed in Table S6.

### Plant materials

Two cultivars of lettuce (*L. sativa*), one exhibiting late bolting and the other early bolting, were selected for investigating the genetic basis of bolting time. These parental lines were crossed to generate an F₂ population suitable for segregation analysis, and the subfamily designated LB356 was utilized in subsequent map-based cloning of *LBL3*. From this family, individuals with extreme phenotypes, classified as LB3-early and LB3-late, were identified and used for further analysis. All plant materials were grown under controlled greenhouse conditions at Henan Agricultural University in Zhengzhou, China, with a 16-hour light/8-hour dark photoperiod maintained at a constant temperature of 26°C. Evaluation of the bolting phenotype under standardized controlled environment conditions revealed high heritability and stability across independent cultivation cycles.

### RT-qPCR and quantification of GA

Total RNA was isolated using RNAiso Plus (Takara, Japan) according to the manufacturer’s instructions. cDNA was generated from equal amounts of RNA using the TransScript cDNA Synthesis SuperMix (TransGen Biotech, China) with an Oligo-dT_18_ primer to ensure enrichment of polyadenylated transcripts. Reverse transcription quantitative polymerase chain reaction (RT-qPCR) was carried out following previously established protocols [49], with amplification efficiency and specificity confirmed through melting curve analysis. The expression levels were normalized to the constitutively expressed reference gene *Ubiquitin* (*LsUBQ4*, LG4362429). The corresponding tissues during the rosette and bolting/flowering stages were used for RT-qPCR. All experiments included four independent biological replicates, each assayed in technical replicates, to ensure statistical reliability and reproducibility of the data. The shoot apex tissues at rosette and bolting/flowering stages were used for GA quantification, following a previously published method [50].

### Plasmid construction

To generate complementation constructs, the full-length *LsOFP6* gene was amplified by PCR from cDNA of the early bolting plants. The resulting PCR fragments were cloned into the SacI-digested pl2b-GFP vector. For CRISPR/Cas9-mediated knockout experiments, sgRNAs specifically targeting *LsOFP6*, *LsCOL3*, or *LsTSF1* were designed and inserted into the corresponding CRISPR/Cas9 binary vectors. In the overexpression assay, the coding sequences (CDS) of *LsOFP6*, *LsTSF1*, and *LsTFL1* were individually amplified and integrated into the StuI-digested pl2b-GFP vector under the control of the *LsUBQ4* promoter to ensure constitutive expression. A GFP-tag was added in-frame at the 3′ end of each target gene to generate C-terminal fluorescent fusion proteins. The *Agrobacterium* (strain EHA105) transformants carrying the correct constructs were selected and used for subsequent plant transformation assays.

### Promoter activity analysis

The dual-luciferase reporter assay was executed following the manufacturer’s protocol (Promega). We PCR-amplified promoter regions (~2000 bp) of the *LsTSF1* and *LsTFL1* genes and individually cloned each fragment into the pGreenII0800-LUC vector upstream of the firefly luciferase gene (*LUC*) open reading frame. Within the same vector system, the *Renilla* luciferase reporter (*REN*), under the control of the constitutive CaMV 35S promoter, served as the internal transfection control. These recombinant constructs were transiently delivered into *N. benthamiana* leaves via *Agrobacterium* infiltration. The luciferase activity data in this study are based on at least four independent biological replicates. Fluorescence intensities for both LUC and REN were quantified 48-hour postinfiltration using a Tecan Infinite 200 PRO luminometer.

### Bimolecular fluorescence complementation

Bimolecular fluorescence complementation (BiFC) assays were conducted according to established methods [48]. The full-length coding sequences of *LsCOL3* and *LsOFP6* were inserted into the pSPYNE(R)173 and pSPYCE(M) vectors, respectively. The constructs 35S:LsOFP6-eYFP_C155_ and 35S:LsCOL3-eYFP_N173_ were co-expressed in *N. benthamiana* leaves for 48 hours, after which eYFP fluorescence was detected using a laser-scanning confocal microscope (Leica TCS SP5).

### Y1H and Y2H

For yeast one-hybrid (Y1H) assays, DNA fragments corresponding to the P1, P2, and P3 regions of the *LsTSF1* promoter, and the P4, P5, and P6 regions of the *LsTFL1* promoter, were individually amplified and inserted into the XhoI-linearized bait vector (pAbAi). The coding sequence of *LsCOL3* was cloned into the EcoRI/BamHI-digested prey vector (pGADT7). The bait vectors were linearized with BbsI and co-transformed with the prey construct into Y1H Gold yeast cells. Transformants were first selected on SD/−Leu/−Ura medium, followed by screening on SD/−Leu/−Ura/AbA plates containing 500 ng/ml aureobasidin A. Negative controls consisted of yeast co-transformed with p53-AbAi and empty pGADT7, while positive controls contained p53-AbAi and pGADT7-p53.

For yeast two-hybrid (Y2H) analysis, the coding sequence of *LsOFP6* was cloned into the bait vector pGBKT7, and that of *LsCOL3* into the prey vector pGADT7. The two constructs were co-introduced into Y2H yeast, and interactions were selected on SD/−Leu/−Trp/−Ade/−His medium. All primers for Y1H and Y2H experiments are listed in Table S6.

### Electrophoretic mobility shift assay

The electrophoretic mobility shift assay (EMSA) was carried out following the manufacturer’s guidelines (Thermo Fisher Scientific). The MBP-LsOFP6 fusion protein was expressed and purified for the assay. For binding reactions, 3 μl of purified MBP-LsOFP6 protein was incubated with 20 pmol of FAM-labeled probes. Probes derived from the *LsTSF1* promoter included P2-a, P2-b, and P2-c; probes from the *LsTFL1* promoter included P4-a, P4-b, and P4-c. The MBP protein alone was used as a negative control. Fifty-fold unlabeled probe relative to the labeled probe was used for the competition assays. The probe sequences are listed in Table S6.

## Supplementary Material

Web_Material_uhag225

## Data Availability

All sequencing datasets are available in the National Center for Biotechnology Information (NCBI) database under BioProject ID PRJNA1382769. The sequence of the *LsOFP6*, *LsCOL3*, *LsTSF1*, and *LsTFL1* genes are available in GenBank (LOC111912164, LOC111894909, LOC111907824, LOC111903054).
