# Supplementary material for: OVATE family protein 6 interacts with CONSTANS-LIKE 3 to regulate bolting by transcriptional control of FLOWERING LOCUS T/TERMINAL FLOWER 1 family genes in lettuce
Source: Hortic Res. 2026 Jun 5;13(11):uhag225. doi: 10.1093/hr/uhag225 (PMC13722875; doi:10.1093/hr/uhag225)
Supplement: Supplementary file 1 [file web_material_uhag225.zip › OFP_Bolting_SupplementaL_figures_20260421.docx]

## Supplementary figures and figure legends

**Figure S1.** The bolting time phenotypes of two parents and the F_2_ population.

(A) The front view of two parents, early bolting parent (left) and late bolting parent (right). Bar = 10 cm.

(B) The bolting time of two parents. Significant differences were determined by *t*-test (** for *p* < 0.01). DAS represents ‘Days after sowing’.

(C) The individual number in the F_2_ population categorized by different bolting time.

**Figure S2.** LsOFP6-mediated bolting regulation is independent of GA pathway in the *Lsblh2* *Lskn1* background.

(A–D) The GA1 content in the LB3-early (*LsOFP6*) plants and LB3-late (*Lsofp6*) plants at rosette stage (A), transition stage (B), bolting stage (C), and flowering stage (D). Data are shown as means ± SD of four biological replicates (two-tailed Student’s *t*-test, ns for no significance).

(E–H) The GA3 content in the LB3-early (*LsOFP6*) plants and LB3-late (*Lsofp6*) plants at rosette stage (E), transition stage (F), bolting stage (G), and flowering stage (H). Data are shown as means ± SD of four biological replicates (two-tailed Student’s *t*-test, ns for no significance).

(I–L) The relative expression of the *LsGA20ox1* in the LB3-early (*LsOFP6*) plants and LB3-late (*Lsofp6*) plants at the rosette stage (I), transition stage (J), bolting stage (K), and flowering stage (L). Data are shown as means ± SD of four biological replicates (two-tailed Student’s *t*-test, ns for no significance).

(M–P) The relative expression of the *LsGA2ox8* in the LB3-early (*LsOFP6*) plants and LB3-late (*Lsofp6*) plants at the rosette stage (M), transition stage (N), bolting stage (O), and flowering stage (P). Data are shown as means ± SD of four biological replicates (two-tailed Student’s *t*-test, ns for no significance).

(Q–T) The relative expression of the *LsGA2ox4* in the LB3-early (*LsOFP6*) plants and LB3-late (*Lsofp6*) plants at the rosette stage (Q), transition stage (R), bolting stage (S), and flowering stage (T). Data are shown as means ± SD of four biological replicates (two-tailed Student’s *t*-test, ns for no significance).

**Figure S3.** The neighbor-joining phylogenetic tree generated by the amino acid sequences of CONSTANS-LIKE proteins from *Lactuca sativa* and Arabidopsis. Numbers on nodes refer to the bootstrap value.

**Figure S4.** The amino acid sequence alignment of LsTSF1 (LG2192922) and FT (AT1G65480).

**Figure S5.** The amino acid sequence alignment of LsTSF1 (LG2192922) and TSF (AT4G20370).

**Figure S6.** Schematic diagram of the effectors and reporters used in the dual-luciferase assays.

**Figure S7.** The expression of *LsOFP6* and *LsCOL3* genes and their correlation analysis in the lettuce natural population.

(A) The expression of *LsOFP6* and *LsCOL3* genes in the lettuce natural population.

(B) The expression correlation analysis between *LsOFP6* and *LsCOL3* in the lettuce natural population.
